# Enhancement in the Catalytic Properties of CotA Laccase from *Bacillus pumilus* via High-Throughput Screening Using Malachite Green as a Pressure

**DOI:** 10.3390/microorganisms13020377

**Published:** 2025-02-09

**Authors:** Xiufang Li, Jieru Tian, Xidong Ren, Junming Wang, Xinli Liu

**Affiliations:** 1 State Key Laboratory of Biobased Material and Green Papermaking, Qilu University of Technology, Shandong Academy of Sciences, Jinan 250353, China; 18394391461@163.com (X.L.); t13791072920@163.com (J.T.); renxidong1986@126.com (X.R.); 2 Shandong Provincial Key Laboratory of Microbial Engineering, Department of Bioengineering, Qilu University of Technology, Shandong Academy of Sciences, Jinan 250353, China

**Keywords:** laccase, *Bacillus pumilus*, high-throughput screening, catalytic properties

## Abstract

Bacterial laccase exhibits substantial application potential in various fields. In this study, we constructed a mutation library of CotA laccase from *Bacillus pumilus* using error-prone PCR, and we performed four rounds of enrichment screening under malachite green (MG) pressure. The results demonstrated that the proportions of the four selected mutant strains were significantly increased. The enzyme activities of the four final mutants PW2, PW5, PW4G, and PW6 were 94.34, 75.74, 100.66, and 87.04 U/mg, respectively, representing a significant increase of approximately 2- to 3-fold compared to the wild-type CotA laccase. Notably, PW4 exhibited significantly improved thermal stability at 90 °C and pH tolerance at pH 12.0. Homology modeling analysis revealed that alterations in the amino acid sequence rendered the spatial structure of the enzyme’s catalytic site more favorable for substrate binding. For instance, the substitution of T262A in PW2 and V426I in PW4 shortened the side chains of the amino acids, thereby enlarging the substrate-binding cavity. The G382D mutation in PW2 and PW5 may induce altered protein conformation via spatial steric hindrance or electrostatic interactions, consequently impacting enzyme activity and stability. These findings provide valuable insights for enhancing the industrial application of bacterial laccase.

## 1. Introduction

Laccase (EC 1.10.3.2) is widely acknowledged as an ideal catalyst candidate due to its unique catalytic properties. The compound efficiently catalyzes the oxidation of both phenolic and non-phenolic substrates in an oxygen-rich environment, yielding pure water as the sole byproduct [1] (pp. 461–476). The versatility of laccase is demonstrated by its wide-ranging applications, such as the decolorization of effluents in the textile industry [2], the oxidative degradation of polycyclic aromatic hydrocarbons in contaminated soils [3], environmental pollution monitoring [4], and the improvement of bread’s texture [5]. Fungal laccases exhibit a broad substrate spectrum due to their elevated redox potential. However, the enzymatic activity of these proteins is limited to a relatively narrow range of conditions: specifically, pH values between 3.5 and 5.5, and temperatures ranging from 30 to 55 °C [6]. The CotA laccase from *Bacillus subtilis* has garnered considerable attention in both academic research and industrial applications due to its exceptional stability and remarkable catalytic efficiency [2,7]. The CotA laccase from *Bacillus licheniformis*, *B. pumilus*, and *Bacillus altitudinis* also exhibited considerable potential for application in challenging environments (90 °C, pH 3.0–10.0, and organic solvents) [8,9,10]. The *cotA* gene sequences from *B. pumilus* W3 and *B. subtilis* share 67.98% sequence homology. Studies have demonstrated that the CotA laccase from *B. pumilus* can effectively degrade recalcitrant dyestuffs, achieving decolorization rates of 52.64% for Reactive Black and 76.59% for Congo Red (CR) within 24 h [11,12]. This highlights the significant environmental application potential of this enzyme.

Wang et al. achieved a laccase activity of 81.4 ± 1.9 U/mg in *Bacillus amyloliquefaciens* by introducing a targeted D501G mutation. However, under elevated temperature conditions, the mutant exhibited a 33.3% reduction in performance, which represents an undesirable outcome [13]. Li et al. conducted targeted mutagenesis to convert the hydrophobic amino acid residue P455 to a hydrophilic one (P455S), leading to an 80% increase in the activity of CotA laccase from *B. subtilis* [14]. Xu et al. enhanced the catalytic efficiency of the CotA laccase double mutant S208G/F227A through rational modification of key amino acids. However, this modification came at a cost, as the half-life of the mutant was reduced to one-third of that of the wild-type enzyme at 70 °C [9]. Although rational modification techniques have led to some progress in enhancing the catalytic attributes of bacterial laccase, the desired outcome has not yet been achieved. Current studies on the CotA laccase from *B. pumilus* have primarily focused on the rational modification of key amino acids within the catalytic region. The random modification strategy, coupled with high-throughput screening technology, was utilized to evaluate a diverse library of mutants. This approach not only complements and refines the rational design and optimization of laccase but also establishes a robust theoretical foundation for further enhancements in laccase enzyme activity [15,16,17].

The present study employed a liquid high-throughput screening to convert the intracellular laccase enzyme activity, which is challenging to detect, into a growth rate that can be easily discerned. After successive enrichment screenings, four mutant laccases exhibiting significantly improved catalytic activity and enhanced stability were obtained. Finally, we conducted a theoretical speculation regarding the enhancement of enzyme activity by employing homology modeling analysis.

## 2. Materials and Methods

### 2.1. Strains and Plasmid

The gene encoding the putative multicopper oxidase CotA from *B. pumilus* W3 (KF040050.1) was codon-optimized and synthesized by General Biol (Anhui) Co., Ltd. (Chuzhou, China). The plasmid pET28a was preserved in our laboratory. All strains were cultured in Luria–Bertani (LB) medium (HuanKai Microbial, Guangzhou, Guangdong, China). The M9 inorganic salt medium was employed for the high-throughput screening of mutants (1 L: 30.0 g of glucose, 15.2 g of Na_2_HPO_4_·12H_2_O, 3.0 g of KH_2_PO_4_, 0.5 g of NaCl, 1.0 g of NH_4_Cl, 2.0 mL of MgSO_4_ (1.0 M), 1.0 mL of CaCl_2_ (0.1 M), pH 7.0). LBPG medium was used for the induction culture of laccase (1 L: 5 g of yeast extract, 10 g of tryptone, 4 g of glucose, 15.2 g of Na_2_HPO_4_·12H_2_O, 3.0 g of KH_2_PO_4_, pH 7.2).

### 2.2. Construction of the Mutant Libraries of CotA Laccase

The mutant libraries were generated by error-prone PCR using pET28a-*cotA* as the template, with a forward primer (5′-GAATTCATGAACCTGGAAAAATTCGTTGAT-3′) and reverse primer (5′-GCGGCCGCTTACTGAATAATATCCATCGGACGC-3′), via the GeneMorph II Random Mutagenesis Kit (Agilent Technologies, Santa Clara, CA, USA). The plasmid pET28a was digested with EcoRI and NotI, and then the epPCR product was ligated using the ClonExpress II one-step cloning kit (Nanjing Vazyme Biotech, Nanjing, China). The recombinant plasmids were subsequently transformed into competent cells of *E. coli* BL21 to establish a CotA laccase mutant library. The mutation database was enriched by constructing six parallel mutation libraries.

### 2.3. Construction and Application of a High-Throughput Screening System for Mutant Screening

The stressor MG was chosen for implementation in the high-throughput screening system, and a single-factor experiment was devised to optimize the screening conditions. The variables investigated included the concentration of MG (12, 14, 16, and 18 mg/L), the initial inoculum (OD_600_ = 0.1, 0.15, 0.2, and 0.3), and the isopropyl-beta-D-thiogalactoside (IPTG) concentration (0.2, 0.4, 0.6, and 1.0 mM). According to the aforementioned conditions, the activated recombinant strain harboring the *cotA* gene was inoculated into 50 mL of M9 medium supplemented with 25 μg/mL kanamycin (Kan), and the optimal combination of screening conditions was determined by monitoring the growth dynamics of the strain. The pET28a plasmid-containing strain was employed as the control.

Subsequently, mutant libraries were eluted from solid medium with 1 mL of saline and inoculated at OD_600_ = 0.2 into 50 mL of M9 medium (14 mg/L MG, 0.4 mM IPTG, 25 μg/mL Kan) enriched to OD_600_ = 1.2–1.5 (37 °C, 200 rpm). For each completed round of enrichment, the plasmids in the culture were extracted and retransformed into *E. coli* BL21. Concurrently, the culture from each round of enrichment was coated on LB solid medium and incubated for 16 h at 37 °C. Single colonies were then randomly selected for DNA sequencing to analyze the proportion of each mutant in the colony. The effectiveness of the system for enriching mutants was assessed by examining the growth rate of the mutants under screening conditions. The wild-type strain was employed as the control. All experiments were performed in triplicate.

### 2.4. Recombinant Laccase: Heterologous Expression and Purification

The modified mutants were incubated in LB medium (25 μg/mL Kan) for 12 h at 37 °C and 200 rpm. The pre-culture was inoculated into 50 mL of LB medium (25 μg/mL Kan) at a dilution ratio of 1:50 (*v*/*v*) and incubated until the optical density at 600 nm reached 0.4–0.5. The expression of CotA laccase was induced by adding IPTG and CuSO_4_ at final concentrations of 0.4 mM and 0.25 mM, respectively. The bacteria were cultured in a shake flask at 25 °C for 6 h at 150 rpm, followed by a quiescent period of 10 h. The cultures were subsequently centrifuged at 8000 rpm for 10 min at 4 °C. The bacteria were harvested and washed twice with PBS (pH 7.4), and then resuspended in a buffer solution containing 20 mM PB, 0.5 M NaCl, and 10 mM imidazole at pH 7.4. The cells were disrupted by sonication on ice at 40% power for 15 min, and the resulting supernatant was collected by centrifugation (10,000 rpm, 4 °C, 20 min) and filtered through a 0.22 μm membrane.

The enzyme liquids were subjected to purification using nickel column affinity chromatography for refinement. The buffers used in this study included a binding/washing buffer (pH 7.4, 10–30 mM imidazole) and an elution buffer (pH 7.4, 250–500 mM imidazole) [9]. Following dialysis, the molecular weight of the CotA laccase protein was determined using 10% SDS-PAGE, while the concentration of the purified CotA protein was assessed utilizing a modified Bradford Protein Assay Kit (Beyotime Biotechnology, Shanghai, China) [18].

### 2.5. Assessment of Enzymatic Activity

Laccase activity unit (U): the amount of laccase necessary to completely oxidize 1 µmol of ABTS within a minute. The laccase activity was assessed by monitoring the absorbance change at 420 nm in the enzyme reaction mixture over a duration of 3 min, with measurements taken every 30 s. ABTS was employed as the substrate for the enzymatic reaction [12]. The kinetic parameters K_m_ and k*_cat_* were determined at 50 °C and pH 3.5 using ABTS as the substrate, with concentrations ranging from 0.01 to 1 mM. The resulting parameters were subsequently fitted to the Michaelis–Menten equation [19]. The determination was repeated thrice for each sample. The enzyme activity was calculated utilizing the following formula:

(1) $$\mathrm{enzyme}\;\mathrm{activity}\;(U\;L^{-1})=\frac{∆OD\times V_{T}\times 10^{6}}{∆T\times V_{E}\times 36,000}\times N,$$

where ΔOD represents the difference in absorbance at 420 nm, V_T_ denotes the total volume of the reaction system, N is the dilution ratio of the enzyme solution, ΔT is the total reaction time, V_E_ signifies the volume of the enzyme solution within the system, and ε is the molar extinction coefficient.

### 2.6. Comprehensive Analysis of Enzymatic Properties of CotA Laccase

To determine the optimal temperature for the mutant CotA laccase, experiments were conducted at pH 3.5, starting at 30 °C and increasing the temperature in 10 °C increments up to 90 °C. To assess the impact of high temperatures on the stability of enzyme activity, enzyme solutions were incubated at 60 °C for 6 h, with enzyme activity measured at hourly intervals. Additionally, enzyme solutions were incubated at 90 °C for 1 h, with enzyme activity assessed every 10 min. ABTS was used as the substrate to evaluate the residual enzyme activity at 50 °C and pH 3.5. The pH of the enzyme reaction mixture was systematically adjusted from 2.5 to 7.0 in 0.5 increments using citrate and phosphate buffers, and the effect of pH on laccase activity was rigorously evaluated. Furthermore, to evaluate the stability of laccase under varying pH conditions, the enzyme solution was incubated in buffers with pH values ranging from 3.0 to 12.0 for 10 h at 4 °C. The residual enzyme activity was evaluated at 50 °C and the optimal pH, with ABTS serving as the substrate. The maximum enzyme activity for each laccase was defined as 100% of the corresponding relative enzyme activity.

To investigate the effects of metal ions on laccase activity, we selected eight metal ions: Cu^2+^, Co^2+^, Fe^2+^, K^+^, Mn^2+^, Mg^2+^, Na^+^, and Zn^2+^. These ions were tested at four concentration levels: 2.5 mM, 5.0 mM, 7.5 mM, and 10.0 mM. The impact of these metal ions on laccase activity was systematically evaluated by incubating a mixture of 100 μL of each metal ion solution and an equal volume of purified enzyme solution at 50 °C for 30 min. The enzyme activity of the solution, prepared by mixing the enzyme with deionized water, was set as the 100% reference for determining relative enzyme activity. The purified enzyme solution was incubated with a mixture of 30% (*v*/*v*) ethanol, methanol, acetone, and dimethyl sulfoxide (DMSO) individually for 30 min at 4 °C. The enzyme activity after incubating the enzyme solution in deionized water was set as the 100% reference for relative enzyme activity. The residual activity was evaluated using ABTS as the substrate under conditions of 50 °C and pH 3.5.

Reactive Brilliant Blue X-BR (X-BR), CR, and MG were selected as representative dyes to evaluate the decolorization capacity of the CotA laccase. A standard reaction system was established (3 mL): 25 mg/L dye, 1 U/mL enzyme activity, 0.5 mM ABTS, and 100 mM (pH 9.0) citrate–phosphate buffer, and the decolorization process was conducted at a constant temperature of 50 °C and 100 rpm for 5 h. The absorbance was measured at specific wavelengths (λ_X-BR_ = 603 nm, λ_CR_ = 497 nm, λ_MG_ = 617 nm) using spectrophotometry both before and after the reaction, and the decolorization activity [(A − A_0_)/A] × 100% was calculated (where A_0_ denotes the initial absorbance value, and A denotes the final absorbance value of the reaction). All results presented are the averages derived from three independent experiments.

### 2.7. Homology Modeling Analysis of Mutant CotA Laccases

The *B. subtilis* CotA laccase with a homology of 67.98% (PDB code: 1GSK) was selected as the template to construct a three-dimensional model for the CotA mutants from *B. pumilus* W3, which was generated using the Swiss-Model online server (https://swissmodel.expasy.org/, accessed on 9 October 2024). The mutant CotA models were subsequently visualized and analyzed using PyMOL 2.5.

### 2.8. Statistical Analysis

To ensure the reliability and reproducibility of the experimental results, each experiment was conducted in triplicate. Statistical analysis was performed using SPSS Statistics 26 software. A one-way ANOVA was conducted, followed by Tukey’s HSD post hoc test to assess significant differences among the groups (*p* ≤ 0.05).

## 3. Results and Discussion

### 3.1. Construction and Functional Validation of a High-Throughput Screening System

Organic dyes exhibit an inhibitory effect on cell growth; however, host bacteria that express laccase demonstrate enhanced cell survival or increased growth rates [9,20,21,22]. This property adeptly converts the challenging-to-detect intracellular enzyme activity into an easily measurable cell growth rate [23,24]. Notably, the growth of *E. coil* BL21 remained unaffected even at a concentration as high as 120 mg/L for both CR and X-BR. MG demonstrated a significant inhibitory impact on the growth of *E. coli* BL21.

**Figure 1:**
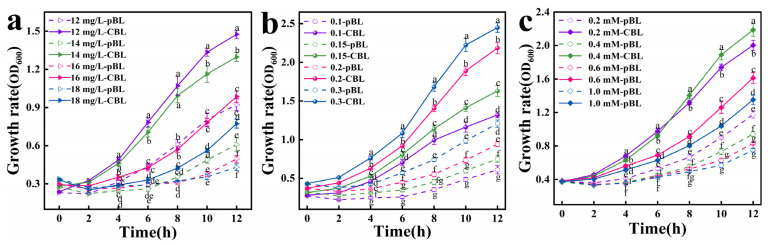
Growth of strain CBL harboring the *cotA* gene (solid line) and strain pBL harboring the plasmid pET28a (dashed line) in M9 medium supplemented with varying concentrations of MG (**a**), different inoculum amounts (**b**), and various IPTG concentrations (**c**): (**a**) Growth of strains in varying concentrations of MG at an IPTG concentration of 0.4 mM and an inoculum density of OD_600_ = 0.1. (**b**) Growth of strains inoculated with varying amounts of inoculum at 0.4 mM IPTG and 14 mg/L MG. (**c**) Growth of strains in varying concentrations of IPTG at a constant MG concentration of 14 mg/L and an inoculum density of OD_600_ = 0.2. The results, derived from three independent experiments, are expressed as the mean ± standard deviation (mean ± SD). Different letters denote the statistical significance (*p* ≤ 0.05) of the effects of MG concentration (**a**), inoculum volume (**b**), and IPTG concentration (**c**) on cell growth at specific timepoints.

To prevent strains from developing adaptive mutations to MG, which could complicate the isolation of dominant mutants, an enrichment step was completed upon reaching a culture OD_600_ of 1.2 to 1.5 [25]. Following the completion of each enrichment round, plasmids were extracted from the culture and subsequently retransformed into *E. coli* BL21. As illustrated in Figure 1a, under the initial conditions of 0.4 mM IPTG and an inoculum density (OD_600_) of 0.1, both strain CBL (harboring the *cotA* gene) and strain pBL (harboring the pET28a plasmid) exhibited a decreasing growth rate as the MG concentration increased. However, the growth rate of CBL was consistently and significantly higher than that of pBL. Specifically, at 14 mg/L MG, the growth rate of CBL was marginally lower compared to that at 12 mg/L, whereas the growth rate of pBL showed a marked reduction. The most pronounced difference in growth rates between CBL and pBL was observed at an MG concentration of 14 mg/L, with CBL exhibiting an average growth rate 1.83 times higher than that of pBL.

As illustrated in Figure 1b, under conditions of 14 mg/L MG and 0.4 mM IPTG, both CBL and pBL demonstrated an increasing trend in their growth rates as the inoculum amount increased. CBL demonstrated markedly higher growth rates when inoculated at an OD_600_ of 0.2 compared to other inoculation densities. Under this condition, the disparity in growth between CBL and pBL was most pronounced, with the average growth rate of CBL being 2.32-fold higher than that of pBL. As illustrated in Figure 1c, under conditions of 14 mg/L MG and an initial inoculum density (OD_600_) of 0.2, the growth rates of both the CBL and pBL strains exhibited a decreasing trend as the IPTG concentration was increased from 0.2 to 1 mM. This phenomenon can be attributed to the cytotoxic effects of high IPTG concentrations on cellular viability [26]. At an IPTG concentration of 0.2 mM, while CBL demonstrated the fastest growth rate, the difference in growth rates between CBL and pBL was only marginal. At an IPTG concentration of 0.4 mM, the growth rates of CBL and pBL showed significant differences, with CBL exhibiting an average growth rate 2.4 times higher than that of pBL. The final optimized conditions were 14 mg/L MG, an inoculum amount of OD_600_ = 0.2, and 0.4 mM IPTG, under which the average growth rate of CBL was 2.4 times higher than that of pBL. The aforementioned results validate the efficacy and reliability of the liquid high-throughput screening system developed in this study.

### 3.2. Efficient Screening and Activity Assessment of Laccase Mutants

**Figure 2:**
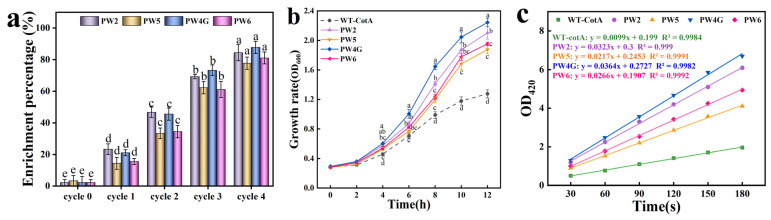
(**a**) Proportion of mutants in each round of enrichment cultures. (**b**) Growth of the wild-type strain (WT-CotA) and mutant strains (PW2, PW5, PW4G, and PW6) was evaluated in M9 medium supplemented with 14 mg/L MG, an initial inoculum density of OD_600_ = 0.1, and induced with 0.4 mM IPTG. (**c**) Linear regression analysis of enzyme activity assays for WT-CotA and the mutants PW2, PW5, PW4G, and PW6. Results were derived from three independent experiments and are expressed as the mean ± SD. Different letters denote statistically significant differences (*p* ≤ 0.05) in enrichment efficiencies among rounds (**a**), as well as significant differences in growth rates between wild-type strains and mutants at specific timepoints (**b**).

We constructed six parallel mutation libraries. The mutants exhibiting laccase activity demonstrated enhanced survival and growth, with a positive correlation between enzyme activity and cellular proliferation rate. Gene sequencing was conducted on the enriched cultures from each round to determine the proportion of each mutant present. Following four consecutive rounds of targeted enrichment, the proportions of the mutants PW2, PW5, PW4G, and PW6 in the colony increased to 85%, 76.67%, 88.33%, and 78.33% from their original frequencies of only 1.67%, 5%, 5%, and 3.33%, respectively (Figure 2a). Subsequently, the growth rate of the mutants was quantified in M9 medium supplemented with MG (Figure 2b). The strain harboring the mutant *cotA* gene exhibited a significantly enhanced growth rate in comparison to the wild-type strain. Among the mutants, PW4G displayed the highest growth rate (0.187 h^−1^), which was 1.83-fold higher than that of the wild-type strain. Four mutants and their mutation sites are detailed in Table 1. Following SDS-PAGE analysis, all purified mutants exhibited a molecular weight of approximately 65 kDa (Figure S1), which is consistent with findings reported in previous studies [27].

The kinetic parameters (K_m_ and k*_cat_*) and specific enzymatic activities of the purified wild-type CotA laccase and its mutants were evaluated using ABTS as the substrate (Table 2). Compared to the wild-type laccase, the K_m_ values of the mutants ranged from 0.092 to 0.158 mM, representing a significant reduction. Concurrently, both the catalytic efficiency and the specific activity of the mutants exhibited substantial increases, as detailed in Table 2. Specifically, the catalytic efficiency of PW2, PW5, PW4G, and PW6 increased by 5.62-fold, 3.40-fold, 6.08-fold, and 4.42-fold, respectively. The specific enzymatic activities of PW2, PW5, PW4G, and PW6 were 94.34 U/mg, 75.74 U/mg, 100.66 U/mg, and 87.04 U/mg, respectively. These values exhibited a significant increase compared to the wild-type CotA laccase, with fold changes of 2.84-fold (PW2), 2.28-fold (PW5), 3.03-fold (PW4G), and 2.62-fold (PW6). The enzyme activities of PW2 and PW4G were found to exhibit a significant increase of 29.86% and 38.55%, respectively, when compared to the results obtained from previous targeted mutation studies [28].

### 3.3. Enzymatic Characterization of the CotA Laccase

#### 3.3.1. Impact of Temperature Fluctuations on the Enzymatic Activity and Thermal Stability of CotA Laccase

**Figure 3:**
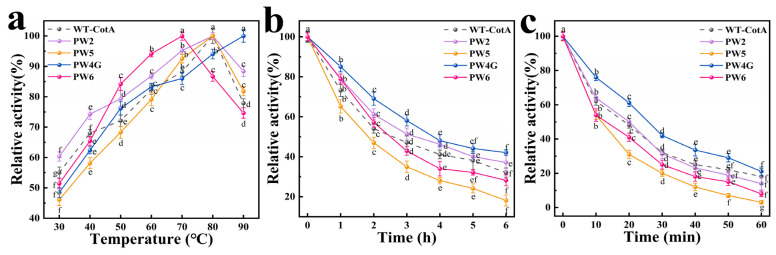
Effects of temperature on activity of the wild-type CotA (WT-CotA) laccase and mutants PW2, PW5, PW4G, and PW6: (**a**) Relative activities of WT-CotA and the mutants PW2, PW5, PW4G, and PW6 across a temperature range of 30 to 90 °C. (**b**) The relative activities of WT-CotA and the mutants PW2, PW5, PW4G, and PW6 were evaluated by incubating them at 60 °C for 6 h. (**c**) The relative activities of WT-CotA and the mutants PW2, PW5, PW4G, and PW6 were evaluated by incubating them at 90 °C for 1 h. The maximum enzyme activity for each laccase was set as the reference point, defined as 100% relative enzyme activity. The residual activity was assessed using ABTS as the substrate at 50 °C and pH 3.5. The results, derived from three independent experiments, are expressed as the mean ± SD. Different letters denote the statistical significance (*p* ≤ 0.05) of the effects of varying temperatures on laccase activity under specific conditions (**a**), as well as the statistical significance of differences in cell growth at designated timepoints (**b**,**c**).

The activity of CotA laccase increased with increasing temperature (Figure 3a). PW6 exhibited the highest enzyme activity at 70 °C, while PW2, PW5, and the wild-type CotA laccase (WT-CotA) demonstrated the highest enzyme activity at 80 °C. The optimal temperature for PW4G was found to be elevated, with the maximum enzyme activity observed at 90 °C. PW4G exhibited good high-temperature stability (Figure 3b,c). Notably, the residual enzyme activities of PW4G remained above 40% after 30 min of incubation at 90 °C. However, the stability of PW5 and PW6 was reduced and the enzyme activity was barely detectable after 1 h of incubation at 90 °C. The maximum enzyme activity for each laccase was defined as 100% of the corresponding relative enzyme activity.

#### 3.3.2. Impact of pH Variation on the Performance and Robustness of CotA Laccase

**Figure 4:**
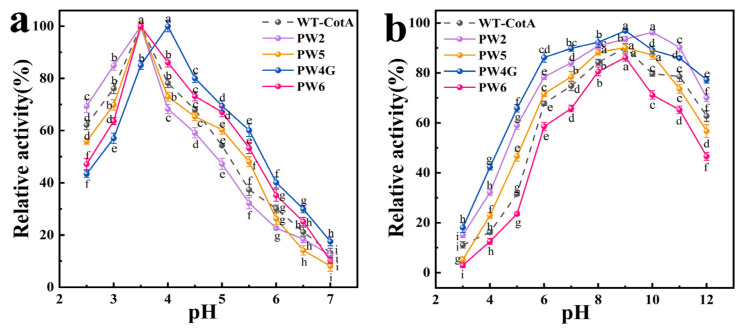
Effects of pH on activity of the wild-type CotA (WT-CotA) laccase and the mutants PW2, PW5, PW4G, and PW6: (**a**) The relative activities of WT-CotA and the mutants PW2, PW5, PW4G, and PW6 were evaluated across a pH range of 2.5 to 7.0. (**b**) The relative activities of WT-CotA and the mutants PW2, PW5, PW4G, and PW6 were evaluated by incubating them at pH levels ranging from 3.0 to 12.0 for 10 h at 4 °C. The maximum enzyme activity for each laccase was set as the reference point, defined as 100% relative enzyme activity. The residual activity was assessed using ABTS as the substrate at 50 °C and the optimal pH. Results were derived from three independent experiments and are expressed as the mean ± SD. Different letters indicate the statistically significant effects (*p* ≤ 0.05) of varying pH values on laccase activity under specified conditions.

To determine the optimal pH for laccase, we measured its relative activity under a range of pH conditions. The enzyme activity of PW2, PW5, and PW6 was found to be highest at pH 3.5, similar to that observed for the WT-CotA. The optimal pH of PW4G exhibited slight fluctuations, with the highest activity observed at pH 4.0 (Figure 4a). Furthermore, the pH stability of laccase was evaluated by measuring its residual relative activity following 10 h of incubation across a range of pH levels. The residual activity of both WT-CotA and the mutants remained above 61% in the pH 6–11 range (Figure 4b). The remarkable stability of PW2 was demonstrated by its high residual enzyme activity of 96.30 ± 1.08% after a 2 h incubation at pH 10.0. It is noteworthy that the residual activity of PW2 and PW4G remained above 70% after incubation at pH 12.0, providing further evidence that these mutant enzymes have enhanced alkaline tolerance properties. The maximum enzyme activity for each laccase was defined as 100% of the corresponding relative enzyme activity.

#### 3.3.3. Effects of Metal Ions on the Enzyme Activity of CotA Laccase

**Figure 5:**
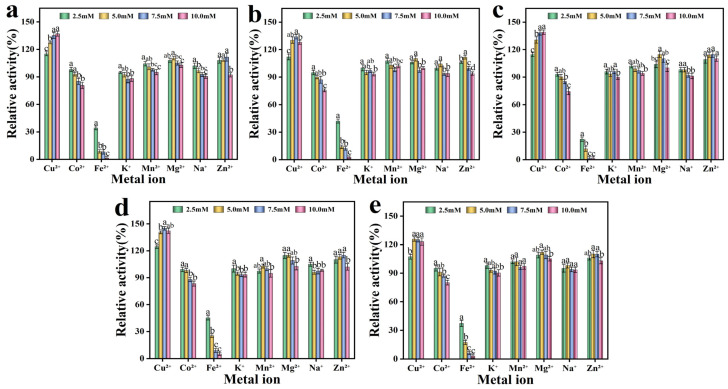
Effects of varying concentrations of metal ions on activity of (**a**) the wild-type CotA (WT-CotA) laccase and the mutants (**b**) PW2, (**c**) PW5, (**d**) PW4G, (**e**) and PW6. The enzyme activity of the solution, prepared by mixing the enzyme with deionized water, was designated as the 100% reference for relative enzyme activity. The results, derived from three independent experiments, are expressed as the mean ± SD. Different letters indicate statistically significant differences (*p* ≤ 0.05) in laccase activity due to varying metal ion concentrations.

The impact of metal ions on the activity of CotA laccase demonstrated considerable variations (Figure 5). Specifically, Cu^2+^, Mg^2+^, and Zn^2+^ exhibited differential enhancement effects on the activity of both WT-CotA and its mutants. Cu^2+^ demonstrated a significant enhancement of laccase activity, with its promotional effects increasing progressively as the concentration rose. Specifically, at a concentration of 7.5 mM, enzyme activity was observed to increase by 25–45%. Copper is an essential component of the laccase active site, and the enhancement of laccase activity by Cu^2+^ can be attributed to its specific occupation of the T1 copper-binding site [29]. In contrast, Co^2+^, K^+^, and Na^+^ exerted a mild inhibitory effect on laccase activity. Notably, Fe^2+^ significantly inhibited laccase activity, reducing it to approximately 35% of the original level at a concentration of 2.5 mM. Furthermore, when the concentration of Fe^2+^ was elevated to 5.0 mM, enzyme activity decreased to approximately 10%. This inhibitory effect is likely attributable to the competition between the substrate and oxidized Fe^3+^ for the T1 Cu-binding site [30]. The enzyme activity of the solution, prepared by mixing the enzyme with deionized water, was set as the 100% reference for relative enzyme activity.

#### 3.3.4. Analysis of Organic Solvent Resistance of CotA Laccase

**Figure 6:**
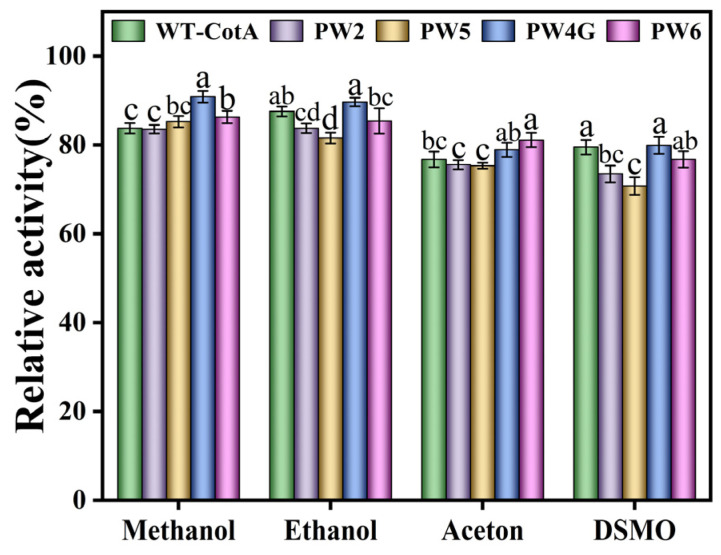
Effects of organic solvents on the activity of wild-type CotA laccase (WT-CotA) and the mutants PW2, PW5, PW4G, and PW6. The enzyme activity measured after incubating the enzyme solution in deionized water was designated as the 100% reference for relative enzyme activity. The results, derived from three independent experiments, are expressed as the mean ± SD. Different letters indicate the statistically significant effects (*p* ≤ 0.05) of organic solvents on the activities of both wild-type and mutant laccases.

The WT-CotA and mutants exhibited excellent solvent tolerance across all tested organic solvents (Figure 6). The PW4G demonstrated a remarkable residual enzyme activity of 90.87 ± 1.31% in a 30% methanol solution, exhibiting an enhancement of 7.1% compared to the wild-type. Additionally, the residual enzyme activity of PW4G was observed to remain above 80% in various other organic solvents. PW6 exhibited the most pronounced tolerance in a 30% acetone solution. The enzyme activity measured after incubating the enzyme solution in deionized water was set as the 100% reference for relative enzyme activity.

#### 3.3.5. Decolorization of Different Dyes by CotA Laccase

**Figure 7:**
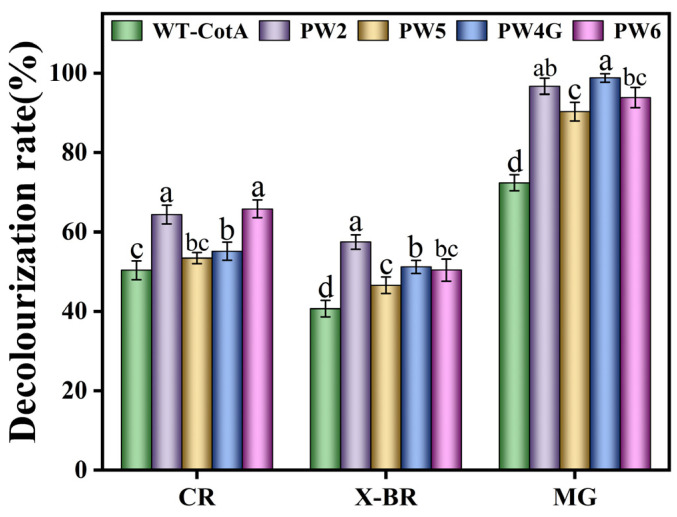
Evaluation of the decolorization efficiency of wild-type CotA laccase and the mutants PW2, PW5, PW4G, and PW6 against X-BR, CR, and MG. Results were derived from three independent experiments and are expressed as the mean ± SD. Different letters denote statistically significant differences (*p* ≤ 0.05) in decolorization rates between the wild-type and mutant laccases.

The mutants exhibited significant decolorization of CR, X-BR, and MG at a concentration of 25 mg/L in the presence of ABTS (Figure 7). Specifically, PW2 and PW4G demonstrated over 96% decolorization of MG within 5 h, representing a significant enhancement of approximately 25% compared to the wild-type laccase. This enhancement represents a significant improvement over the previously reported 6% increase achieved through targeted mutations [9]. After 5 h of PW6 treatment, the decolorization rate of MG reached 93.9 ± 2.5%. Furthermore, PW2 increased the decolorization efficiency of CR and X-BR by 14% and 16.8%, respectively. Furthermore, PW6 enhanced the decolorization efficiency of CR by 15.4%. The results presented herein demonstrate the remarkable efficacy of the mutants in degrading MG, thereby highlighting their substantial practical value.

### 3.4. Homology Modeling Analysis of Mutant CotA Laccase

**Figure 8:**
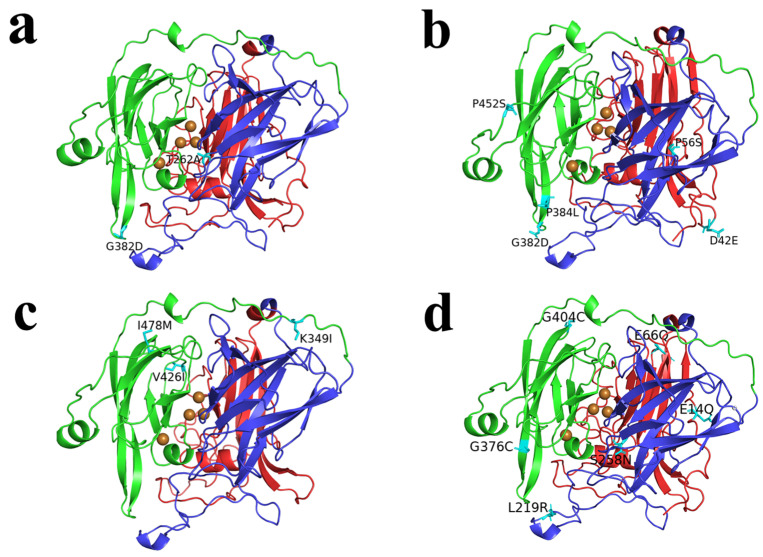
Display of 3D structures and mutant amino acid residues of mutants (**a**) PW2, (**b**) PW5, (**c**) PW4G, and (**d**) PW6. Domain 1: red. Domain 2: blue. Domain 3: green.

To elucidate the mechanisms underlying the altered enzyme activity in the mutant enzymes, we analyzed the impact of amino acid sequence modifications on enzyme function through homology modeling and conducted a preliminary investigation into the associated mechanisms. The three-dimensional structural model of the mutant CotA laccase from *B. pumilus* W3 was constructed using the Swiss-Model online server and analyzed through PyMOL 2.5 visualization to gain insights into the catalytic mechanism (Figure 8). The substitution of T262A near the center of T2Cu in PW2 results in a shorter side chain at amino acid residue 262, thereby altering the local structural environment, creating a more favorable environment for effective substrate binding to the protein [11]. Furthermore, given that Ala is a hydrophobic amino acid, this alteration may also enhance enzyme stability [31]. The G382D mutation in PW2 replaces a small, neutral glycine residue with a negatively charged aspartic acid side chain. This substitution could potentially alter protein conformation via spatial steric hindrance or electrostatic interactions, consequently impacting enzyme activity and stability [32].

The P384L mutations in PW5, located proximal to the active center of T1Cu, could potentially induce conformational alterations in the substrate-binding pocket [33]. The transition of P452S, located on the protein surface, from hydrophobic to hydrophilic characteristics enhances the flexibility of the protein surface. The P452S mutation appears to enhance protein–substrate binding by augmenting the flexibility of the protein [34]. It is worth noting that the same G382D mutation was detected in both PW5 and PW2, implying that G382D might serve as a crucial catalytic site essential for augmenting the enzymatic activity.

The replacement of V426I may lead to an enlargement of the substrate-binding cavity in PW4, thereby enhancing substrate entry and binding efficiency [11]. Furthermore, the substitution of the key amino acid I478M is likely to induce conformational changes in the binding site, which may serve as an additional factor contributing to the enhancement of laccase activity [35]. The substitution of Lys at position 349 and Val at position 426 with Ile resulted in an increased Ile content, which is likely a significant contributor to the enhanced stability of PW4G [36].

The mutations G376C and G404C may facilitate the formation of disulfide bonds within PW6, thereby potentially enhancing the stability of laccase [37]. Enhancing hydrophobic interactions can augment the enzyme’s affinity for its substrate. Therefore, the formation of hydrophobic cystine residues via the G376C and G404C mutations within the pocket is likely to contribute to the enhanced catalytic activity and improved dye decolorization capability of PW6 [12]. The S258N mutation, located in close proximity to the active site, may enhance the catalytic efficiency of the enzyme by facilitating electrostatic interactions or altering hydrogen bond networks [28]. In a high-concentration organic solvent evolution experiment, the T480A mutant, which is located distal to the trinuclear copper cluster, exhibited 2.3-fold higher catalytic activity relative to the wild-type laccase and demonstrated significantly enhanced stability in organic solvents [38]. Mutations located distant from the catalytic site can enhance enzymes’ catalytic efficiency by modulating the local microenvironment or affecting interactions between distal structural domains.

## 4. Conclusions

Bacterial laccases demonstrate exceptional stability, rendering them highly promising for diverse applications across industries and environmental science [2,3]. Nevertheless, the restricted catalytic efficiency and low yield of natural bacterial laccase present substantial obstacles to its broad application. This study innovatively employed MG as both a screening stressor and a catalytic substrate to enrich mutants that exhibited enhanced catalytic activity and efficient decolorization through shake-flask culture. After four rounds of enrichment culture, four mutants that demonstrated a growth advantage under stressful conditions were successfully identified.

The decolorization efficiencies of PW2, PW4G, and PW6 for MG exceeded 94%. Compared with the wild-type CotA laccase, PW6 exhibited a 16% improvement in CR decolorization. These results indicate a promising potential for the mutants in dye decolorization applications. Notably, the enzyme activity of PW4G was enhanced by 3.03-fold, concurrent with a significant improvement in thermal stability. The residual activity of both WT-CotA and the mutants remained above 61% across the pH range of 6 to 11. These properties equip the mutants with enhanced potential for applications in a variety of challenging environments, such as high-temperature settings and highly alkaline industrial wastewater treatment processes. In the future, we will employ modeling analysis to generate combinations of laccase mutation sites based on the four obtained mutants, with the objective of developing mutant laccases that exhibit enhanced catalytic properties. Furthermore, immobilized enzyme technology has emerged as a research hotspot owing to its significant role in enhancing application stability. The immobilization of mutant enzymes could potentially serve as an effective strategy to improve their stability.

## Figures and Tables

**Table 1:** Mutation sites of the four mutants.

Laccase	Mutation Sites of Amino Acids
PW2	T262A/G82D
PW5	D42E/P56S/G382D/P384L/P452S
PW4G	K349I/V426I/I478M
PW6	E14Q/E66Q/L219R/S258N/G376C/G404C

**Table 2:** Wild-type and mutant laccase activities with ABTS as the substrate.

Laccase	K_m_ (mM)	k*_cat_* (s^−1^)	k*_cat_*/K_m_ (s^−1^ mM^−1^) ^1^	Protein Yield (mg/L)	Volumetric Activity (U/L)	Specific Enzyme Activity (U/mg)
WT-CotA	0.255	39.62	155.38	12.17 ± 0.71	404.35 ± 18.95	33.22
PW2	0.103	89.87	872.56	14.35 ± 0.91	1354.06 ± 22.01	94.34
PW5	0.158	83.21	526.64	11.74 ± 0.79	888.96 ± 18.04	75.74
PW4G	0.092	90.75	945.33	14.86 ± 0.87	1495.91 ± 26.41	100.66
PW6	0.123	84.55	687.40	12.53 ± 0.66	1090.61 ± 32.39	87.04

^1^ The kinetic parameters K_m_ and k*_cat_* were determined at 50 °C and pH 3.5 using ABTS as the substrate, with concentrations ranging from 0.01 to 1 mM.

## Data Availability

The original contributions presented in this study are included in the article/Supplementary Material. Further inquiries can be directed to the corresponding authors.

## Supplementary Materials

The following supporting information can be downloaded at: https://www.mdpi.com/article/10.3390/microorganisms13020377/s1, Figure S1: SDS-PAGE of purified CotA laccase. Lane M: Marker (10–170 kDa); Lane 1: wild-type cotA laccase; Lane 2: mutant PW2; Lane 3: mutant PW5; Lane 4: mutant PW4G; Lane 5: mutant PW6.
