# Supplementary material for: Enhancement in the Catalytic Properties of CotA Laccase from Bacillus pumilus via High-Throughput Screening Using Malachite Green as a Pressure
Source: Microorganisms. 2025 Feb 9;13(2):377. doi: 10.3390/microorganisms13020377 (PMC11858407; doi:10.3390/microorganisms13020377)
Supplement: Supplementary file 1 [file microorganisms-13-00377-s001.zip › microorganisms-3463880-supplementary.pdf]

Supplementary Materials

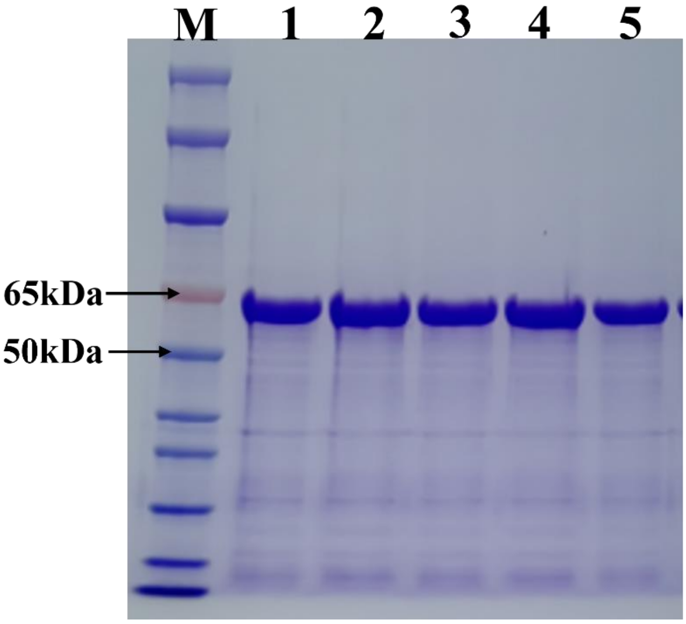

Figure S1. SDS-PAGE of purified cotA laccase. Lane M: Marker (10 - 170 kDa); Lane 1: Wild-type cotA laccase; Lane 2: mutant PW2; Lane 3: mutant PW5; Lane 4: mutant PW4G; Lane 5: mutant PW6.
